# WHAT MOTIVATES PHYSICIANS TO ADDRESS CAREGIVER NEEDS? THE ROLE OF EXPERIENTIAL SIMILARITY

**DOI:** 10.1093/geroni/igac059.2484

**Published:** 2022-12-20

**Authors:** Taeyoung Park, Karl Pillemer, Corinna Löckenhoff, Catherine Riffin

**Affiliations:** Cornell University, Ithaca, New York, United States; Cornell University, Ithaca, New York, United States; Cornell University, Ithaca, New York, United States; Weill Cornell Medical College, New York, New York, United States

## Abstract

Despite the negative emotional and physical consequences of caregiving, caregivers’ needs and risks are often overlooked in health care settings. This study used survey data from a national random sample of primary care physicians (N=106) to examine the factors associated with physicians’ perceived responsibility to identify caregiver needs and risks, focusing on three theoretically implicated variables: 1) experiential similarity (i.e., physicians having personal experience with caregiving), 2) structural similarity (i.e., physicians being older and female), and 3) secondary exposure to caregivers (i.e., more time seeing older patients and a higher percentage of older adults in their patient panel). Physicians in our sample consisted of 42.5% women; most of whom were white (73.6%). The majority (76.5%) agreed or strongly agreed that they were responsible for identifying caregivers’ needs and risks. Multivariable models controlling for physicians’ age and gender revealed that physicians who had personal experience with caregiving were four times more likely than those without caregiving experience to feel responsible to identify caregivers’ needs and risks (adjusted odds ratio [aOR] 3.90; 95% confidence interval [CI] 1.34-11.41) and to assess caregivers’ mental health concerns (aOR 3.58; 95% CI 1.29-9.94). Structural similarity and secondary exposure did not play significant roles in motivating physicians. Findings highlight the role of experiential similarity in physicians’ motivation to assess caregivers’ needs and risks. Future work may benefit from designing intervention programs for physicians that incorporate experiential learning activities (e.g., conversations in which caregivers share their experiences) and evaluating whether such programs enhance physicians’ sensitivity toward family caregivers.

